# 

**Published:** 1902-08-15

**Authors:** 


					﻿THE
DENTAL REGISTER
EDITED BY
NELVILLE S. HOFF, D. D. S.,
ANN ARBOR, MICH.
Vol. LVI.	AUGUST 15, 1902.	No. 8.
PRICE, ONE DOLLAR À-YHAR 1FTADVANCE?
PUBLISHED MONTHLY BY
SAMUEL· A. CROCKER & CO.,
THE OHIO DENTAL AND SURGICAL DEPOT.
to BO "Wr. “tli ^t., Cineiiiniiti, O.
Entered at the Postoffice at Cincinnati, 0., as second-class mail matter.
				

